# European Correspondence

**Published:** 1854-07

**Authors:** Austin Flint

**Affiliations:** Professor of the Theory and Practice of Medicine in the University of Louisville


MEDICINE AND SURGERY.

JULY, 1854. NEW SERIES. VOL. II.—NO. 7.

### ART. I.—EUROPEAN CORRESPONDENCE.

BY AUSTIN FLINT, M. D., PROFESSOR OF THE THEORY AND PRACTICE OF MEDICINE IN THE UNIVERSITY OF LOUISVILLE.

PARIS, May 18, 1854.

*Dear Doctor:*—Of the numerous hospitals of Paris, two are devoted exclusively to cases of *venereal* disease. One is appropriated to male and the other to female patients. The former is called the *Hôpital de Midi*. The buildings were formerly occupied as a monastery. In 1784 it was converted into a hospital for nurses, and new-born infants affected with syphilis. Shortly afterward it became a general syphilitic hospital for persons of both sexes, but subsequently it was reserved for males alone. This hospital contains 336 free beds. It receives, besides, a small number of pay patients. Male attendants are alone employed in this institution. The average number of patients is 3,300.* A physician and two surgeons form the medical staff of the hospital. The physician is Dr. PUCHE; the surgeons are M. M. VIDAL DE CASSIS, and RICORD.

M. RICORD has a world wide reputation as the author of several treatises on syphilitic affections, which have materially altered, in

* For these, and other statistics, I am indebted to that indispensable companion of the stranger in Paris, Galignani's Guide, edition for 1854.

many respects, the pathological views and the practice of the profession of all countries, in this class of diseases. No writer, indeed, for the last ten years, has been so prominently identified, as he, with the literature of this subject. He is emphatically the authority on all points pertaining to it, how justly I am not prepared to say. As a practitioner in this speciality he holds a corresponding position. During his consultation hours his *salons* are crowded with patients of all ages, conditions, and of either sex, who receive from the servant at the door cards numbered in the order of their application, and they are admitted in the same order. It is said that his practice is more lucrative than that of any other practitioner in the city.

The wards of M. Ricord at the *Hôpital de Midi* contain a hundred or more patients, presenting every variety and stage of syphilitic disease. As a field of study for one desirous of devoting attention to this department it is all that could be desired. Here may be observed, at once, groups of cases which in a general hospital, and still more in private practice, would not fall under observation for months or even years. It is in this point of view that Paris offers for clinical studies great advantages. By means of its special hospitals numerous illustrations of the disease pathological conditions pertaining to any particular class of affections, are simultaneously placed before the observer. By this, not only may much be accomplished in comparatively a short space of time, but the advantages of comparison and contrast contribute to accuracy of observation. The difference is like that between prosecuting the study of mineralogy, on the one hand, by collecting specimens as they chance to fall in ones way, examining them and then throwing them aside; and, on the other hand, by resorting to a large and well arranged cabinet.

M. Ricord must be at least fifty years of age; but with an abundance of black hair, a full face free from wrinkles, a brisk gait, and a vivacity of manner amounting almost to boyish friskiness, he appears much younger. Mirthfulness must be a prominent trait in his mental constitution. He passes from bed to bed, greeting his patients always with a smile, often with a jest, and sometimes patting them playfully on the cheek. In these demonstrations of familiarity he compromises somewhat that dignity of demeanor, which is usually preserved by the hospital physicians and surgeons here, without any approach to stiffness or affected pomposity of manner.

After visiting the wards, M. Ricord examines and prescribes for out-patients, who usually apply in great numbers.

The female syphilitic hospital is called the *Hôpital Lourcine.* It contains three hundred beds, of which two hundred and fifty are for adults, and fifty for children. The average number of patients yearly is two thousand. Students and medical practitioners are not admitted to the wards of this hospital without special permission. The medical stranger, however, has no difficulty in obtaining a permit, on application at the *bureau central d'admission.* M. Gosselin, one of the surgeons, had sent me a ticket through a mutual friend, which, however, inadvertantly, I did not carry to the hospital. The *concierge* had no authority to admit me on my own statement of this fact, but he attended me to the ward in which M. Gosselin was just commencing his tour of service, and on introducing myself, I was invited to accompany him. I am not aware that such restrictions exist at any other hospital with the single exception of the *Hôpital de la Maternite.*

M. Gosselin appears to be about forty-five years of age. He is somewhat below the medium stature, rather stout, has a florid complexion, and bright black eyes slightly affected with strabismus. He is one of the very few among the hospital surgeons and physicians who do not appear to be in haste to complete their morning duties. His patients, numbering about one hundred were in two large, well ventilated and very neat wards. One of these wards was assigned to those not requiring examinations with the speculum, cases of constitutional syphilis; the other to cases in which these examinations are required. Connected with the latter ward is a small apartment provided with a speculum table, placed before a window, and here he examined the patients admitted one by one. About thirty patients were examined in this apartment, presenting chancres, vaginitis, vaginal abscesses, buboes, mucous tubercles, ulceration of the neck of the uterus, and intra-uterine inflammation. In one case there existed great enlargement at the labia, and an immense number of vegetations within the labia and around the anus, in several instances he introduced the stick of the nitrate of silver within the uterus. He used the bivalve speculum exclusively, applying in the different cases instruments differing in size. The examination of each patient was attentively made, the vagina, and frequently the rectum being explored with care. As each patient was admitted, an *interne* read the record of the case made at the last examination. During the examination M. GOSSELIN dictated the present record. All this was done deliberately and with careful regard to accuracy. I have not elsewhere met with the same attention to clinical records. M. G. is making a collection of facts probably with reference to a future work. The explanation of this degree of industry which so far as I am able to observe, is unusual at the Parisian hospitals, is perhaps to be found in the fact that M. G. has not yet reached the highest position to which a Parisian surgeon may aspire; and in Paris this position is the reward of talent and labor.

After concluding his examinations of the ward patients, he attended to new cases. In these cases occular examinations were invariably made. Some were admitted into the hospital, and others were placed on the list of out-patients.

Among the inmates of the *Hôpital Lourcine* I observed females of different ages, some appearing as old as fifty, or even more, but much the larger proportion were young. It was painful to see ascend the table for an examination with the speculum a girl so youthful in appearance that M. GOSSELIN was led to inquire her age. Her reply was fifteen years! Many of the patients presented an exterior so respectable that their character would not have been suspected. Others evinced their vocation in their countenances and demeanor. The utmost order and propriety, however, are maintained in the wards; and here, as at most of the hospitals, the nurses are sisters of charity. A card attached to each bed contained among other items, the occupation of the patient. I noticed under this head *domestique*, *lingére*, *couturière*, but in several instances, this space was blank. Probably in the latter cases the persons had deliberately adopted an abandoned life as a profession.

You are, of course, aware that prostitution is practiced in Paris under the sanction of a legal license. A public woman is obliged to inscribe her name in an official register and is kept under the surveillance of the police. She becomes what is technically called *une fille inscrite.* She is compelled to submit to medical examinations, from time to time, and if found diseased she is sent at once to the hospital *Lourcine*, whence she is not discharged except on the certificate of a medical officer of the institution. The subject of legalized prostitution involves important moral considerations which I do not design to discuss. With us the law recognizes the evil only to enact penalties which do not prevent it, if indeed, they tend to diminish it. In France the evil is recognized as one that cannot be prevented by law, and hence that it falls within the scope of legal enactment to mitigate some of its terrible results. It may be doubted whether the system pursued in France and some other countries of Europe operates in any measure as a bounty, for the increase of prostitution. The influence is perhaps the reverse. But as regards the physical consequences to both sexes it is unquestionably true that the regulations so rigidly enforced at Paris tend to mitigate and diminish them in no small degree.

This subject is one of interest and importance to the moralist, and the political economist, as well as to the medical philosopher. It is one of those subjects pertaining to health and happiness which, from a false delicacy, it has been tacitly understood to be either improper or useless to discuss. A late British writer, however, has disregarded this sentiment, and commenced a series of articles in the British and Foreign Medico Chirurgical Review,* which will be likely to call attention to the subject in the United States, as well as in Great Britain. A work published in Paris more than ten years since by A. I. B. PARENT DUCHATELET contains much statistical information respecting the class of abandoned females in Paris. A translation of this work has been, I believe, issued in the United States.

Of each of the thirty-five hospitals and *hospices* of Paris, a majority of which I have already visited, you will hardly expect from me an account. They are all conducted essentially on a similar

* Dr. S. S. HOLLAND. No. of the Review for Jan. I have not seen the subsequent Nos. of the Journal, which probably contain the continuation of the article.

plan, so that, as respects the general arrangement and management, one may be considered to be a type ot the whole. Curiosity, of course, leads the medical visitor in Paris to see most of them; but it is the distinguished members of the profession, more or less of whom are connected with all of them, which render them severally of interest to the stranger. At *La Charité*, for example, at the present time, on a morning's visit one will find VELPEAU, CRUVEILHIER, RAYER, PIORRY, ANDRAL, BOUILLAUD, and BRIQUET examining patients from eight in the morning till ten, each in his respective wards. Here is a list of names which have been conspicuous in medical literature for the last quarter of a century. Clinical lectures are given three times a week by VELPEAU and PIORRY, and clinical *conferences* at the bed-side, by the latter, with his private class, daily. The hospital *Beaujon* possesses interest from the fact that here one may follow BARTH, distinguished as a morbid anatomist and stethoscopist. At the hospital *St Antoine*, the clinical conferences of M. ARAN, a rising member of the profession, will repay for the trouble of a morning excursion. At the children's hospital GUERSANT visits daily, giving clinical lectures and operating twice weekly. On the day of my visit he operated for stone in the bladder, on a boy about ten years old. He performed the bilateral operation, and chloroform was administered freely by means of an inhaler. At *La Pitie*, VALLEIX, author of an admirable work on internal pathology, or the practice of medicine, is one of the physicians now on duty. At the *Hôpital des Cliniques de la Faculté de Medicine*, may be found daily NELATON, the eminent surgical author, one of the most popular of the surgical teachers in Paris, and PAUL DUBOIS not less eminent in the department of midwifery. Clinical lectures are given by both on alternate days. At the *Hôpital Saint Louis*, CAZENAVE, DEVERGIE and HARDY are on service in the medical wards, and clinical courses by all are in progress, consisting of one lecture by each weekly, given on different days. At the same institution MALGAIGNE and DENONVILLIERS are the surgeons visiting daily and giving stated clinical lectures. So at other institutions attractions are derived from the medical officers more or less distinguished, who are connected with them, and who make it an object to render the wards under their control useful to others in the way of medical observation and instruction.

Some of the hospitals, however, in addition to those of which I have given some account, claim special notice. The *Hôpital des Cliniques*, mentioned above, is one of these. This hospital is appropriated to surgical diseases, and cases of midwifery. About one thousand accouchements take place at this hospital during the year. Students of medicine are admitted in sections to practice examinations by the touch, etc., during pregnancy, and to witness the process of parturition. The accoucheur, PAUL DUBOIS, is one of the most prepossessing of the hospital teachers of Paris in his personal appearance, and in his bearing toward his patients, and toward the class who follow him. His clinical lectures are delivered in an easy and agreeable style, relieving the attention of his auditors occasionally with a little quiet humor. NELATON's clinical lectures always draw a full amphitheatre.

The *Hôpital Salpêtriëre*, or *hospice de la vieillesse* possesses great interest, not alone for the medical observer, but for any one not indifferent to the subject of philanthropic institutions. It is appropriated to aged or infirm indigent females, female lunatics and epileptics. This huge establishment contains between five and six thousand inmates. It forms a large community in itself, covering, at a rough estimation, from thirty to fifty acres, pleasantly situated at a short distance from the *Jardin des Plantes.* With this great number of inmates there is no over-crowding. The buildings are ample, containing numerous large, well ventilated rooms which are perfectly neat. The large courts are beautifully arranged with graveled walks running in various directions, groves and shrubbery, so that the aged and feeble inmates may live, as the Parisians of both sexes delight to live, in fine weather, viz: in the open air. It is a truly interesting sight, the groups of paupers seated under the trees, or under a large tent erected to shield them from the sun, with their knitting or sewing, all presenting a tidy appearance and apparently perfectly contented. Such a spectacle redeems Paris from not a little of the much which shocks the moral sense. An examination of the internal arrangements of this institution, the cuisine, the laundry, the pharmaceutic departments, etc., leads one to admire the systematic order and precision, extending to the minutest details, with which the public institutions of this city are conducted. I should not omit to add that connected with the establishment is a large church, ornamented with statuary and valuable paintings, and open, as are all the churches of the Catholic church, at all times. The visitor will here always find numbers of the inmates of the *hospice* engaged in their devotions.

At the present time a highly interesting course of lectures on mental pathology is in progress, by M. BAILLARGER, one of the physicians of the institution. His lectures are delivered on Sunday morning, and among his auditors are many of the *savans* of Paris, in addition to a large class of medical students and practitioners.

The *Hôpital St. Louis* is of incomparable value to one desirous of studying diseases of the skin. I have visited the wards of this institution twice weekly since my arrival in Paris, and shall continue to do so during the summer. I design to devote to it a future letter.

With the great number of hospitals in Paris, situated at remote distances from each other, with clinical lectures going on in all simultaneously, and usually at the same period of the day, viz:—from 7½ A. M. to 10 A. M.; together with the numerous private courses of instruction, and didactic courses at the *Ecole de Medicine*, the *Ecole de Pratique*, the *Jardin des Plantes* and the *sorbonne*, it is entirely out of the question for the medical student to avail himself, at one time, of more than a fractional part of the opportunities constantly offered for prosecuting the medical and collateral sciences. He must select the branches which he desires to pursue, and also make choice of the teachers whose aid he will accept. If he desires to observe the medical, or surgical practice of hospitals, he must, after preliminary examination, elect two or three which he prefers. He must also content himself with a few of the public and private courses of lectures, consoling himself with the reflection that fresh courses on the same subjects are repeated at short intervals, so that he has in prospective those which he is obliged to defer. It is obvious that in order to turn to good account a sojourn in Paris of a few months it is highly important to have certain objects in view, that is, to know what branches one desires to pursue. Without definite ideas on this point, the mind would be likely to be confused, and perhaps discouraged by the multiplicity of advantages. It is for this reason, among others, that one is in a better situation to profit by the facilities which are here presented for scientific studies after having been engaged in the practical duties of the medical profession long enough for one's taste or capacity to take its natural direction, and for a person to know what kind of knowledge he wishes to possess, and to be able to appreciate the opportunities which specially pertain to this city. There are other cogent reasons why it is a measure of very doubtful expediency for a young man to come to Paris to pursue his studies. The temptations to profitless amusements, and vice, are very great, and it must require more than an ordinary stability of character, wholly removed as one is from the restraints of social and domestic influences, to resist the allurements by which he is surrounded. Observation shows that in not a few instances a residence here becomes attractive not so much for its scientific advantages, as for the abundant resources for dissipation.

The hospitals of Paris, of themselves, furnish ample matter for a series of letters; but they form but one class among the many objects which are interesting to the medical observer. There are the school of medicine, the museums, the libraries, the medical journals, the academies and scientific societies, the laws regulating medical education and the medical profession, &c., &c. If my leisure permits, and I can persuade myself that what I may write will prove acceptable to yourself and your readers, you may expect a continued correspondence relating to some of these subjects.

In my first letter I alluded to the fine weather which continued without interruption during the first three weeks after my arrival. I had begun to think that even the elements had been made subservient to the gaiety of *la belle France*, but I have since had occasion to know that a long "*spell*" of bad weather is possible even in Paris. For the past month it has been cold and rainy, with the exception of but a very few days, and on the 20th of May I was writing by a coal fire, which is indispensable to comfort.

Yours truly,

